# Plastid super-barcodes as a tool for species discrimination in feather grasses (Poaceae: *Stipa*)

**DOI:** 10.1038/s41598-018-20399-w

**Published:** 2018-01-31

**Authors:** Katarzyna Krawczyk, Marcin Nobis, Kamil Myszczyński, Ewelina Klichowska, Jakub Sawicki

**Affiliations:** 1 Department of Botany and Nature Protection, Faculty of Biology and Biotechnology, University of Warmia and Mazury in Olsztyn, Olsztyn, Poland; 2 Institute of Botany, Faculty of Biology, Jagiellonian University, Kraków, Poland

## Abstract

Present study was designed to verify which or if any of plastome loci is a hotspot region for mutations and hence might be useful for molecular species identification in feather grasses. 21 newly sequenced complete plastid genomes representing 19 taxa from the genus of *Stipa* were analyzed in search of the most variable and the most discriminative loci within *Stipa*. The results showed that the problem with selecting a good barcode locus for feather grasses lies in the very low level of genetic diversity within its plastome. None of the single chloroplast loci is polymorphic enough to play a role of a barcode or a phylogenetic marker for *Stipa*. The biggest number of taxa was successfully identified by the analysis of 600 bp long DNA fragment comprising a part of *rbcL* gene, the complete *rbcL-rpl23* spacer and a part of *rpl23* gene. The effectiveness of multi-locus barcode composed of six best-performing loci for *Stipa* (*ndhH*, *rpl23*, *ndhF*-*rpl32*, *rpl32-ccsA*, *psbK-psbI* and *petA-psbJ*) didn’t reach 70% of analyzed taxa. The analysis of complete plastome sequences as a super-barcode for *Stipa* although much more effective, still didn’t allow for discrimination of all the analyzed taxa of feather grasses.

## Introduction

Feather grass (*Stipa* L.) is a genus from the tribe of Stipeae (Poaceae), common or dominant in grasslands and steppes in worm temperate regions of the Old World. In narrow concept it comprises over 150 grass species native to Asia, Europe and north Africa^1^. Discrimination of species representing feather grasses is based on their morphological characters and geographical distribution. However, within particular section of *Stipa*, eg. sect. *Smirnovia* Tzvel., sect. *Leiostipa* Dumort., *Barbatae* A. Junge and sect. *Stipa*, there are several couple of taxa that are morphologically very similar to each other and differ in one to few characteristics connected with size of plant or their particular parts, type of indumentum, and/or geographical distribution. As an example of such situation can serve such couple of species like: *Stipa richteriana* Kar. & Kir. – *S. jagnobica* Ovcz. & Czuk.; *S. arabica* Trin. & Rupr. – *S. hohenackeriana* Trin. & Rupr., *S. pennata* L. – *S. borysthenica* Klok., *S. krylovii* Roshev. – *S. sareptana* A.K. Besser; *S. macroglossa* P.A. Smirn. – *S. kungeica* Golosk.; *S. subsessiliflora* (Rupr.) Roshev. – *S. basiplumosa* Munro ex Hook. f.^2–6^. Morphological similarity and variability of particular taxa was a case of taxonomic confusion and discussion within agrologists. Due to high phenotypic plasticity observed within *Stipa*, narrow species concept or taxonomic splitting may cause many difficulties in determination of species, but on the other hand, too broad species concept can also create problems in understanding patterns of diversity^5,7–9^. For precise delimitation of some feather grasses, there is a need to use molecular methods that can help to explain variation that is not masked by phenotypic plasticity and can put new light on hitherto unsolved problems and attempts to trace evolutional tendencies.

Molecular identification of species uses genetic based method called DNA barcoding. The method allows to rapidly identify specimens to species even from trace amounts or degraded sample tissue. DNA barcoding relies on the amplification of specific barcoding locus or multiple loci in the genomes of the target species^10,11^. Numerous loci are applied in plant barcoding with more or less success. Unfortunately, discrimination power of known barcodes is too low to work across all species, especially in higher plants, therefore there is no universal barcode loci neither for all plants nor for grasses^12,13^. To find more robust single- or multi-locus barcode which would increase the resolution in distinguishing among species within particular groups of plants, numerous researches are still ongoing^14^. Despite several researches on *Stipa* carried so far^15–21^, still there is lack of molecular marker which would allow for effective species delimitation within a significant part of *Stipa* representatives nor for resolving phylogenetic relationships within them. For this reason, phylogenetic inferences for *Stipa* are scarce^16^. Thus far researches using molecular methods proved distinctiveness of small group of Himalayan species comprising *Stipa basiplumosa*, *S. capillacea* Keng, *S. penicillata* Hand.-Mazz., *S. purpurea* Griseb., *S. regeliana* Hack. and *S. roborowskyi* Roshev. from remaining *Stipa* species representing almost all distinguished sections within the genus^17,21^. Therefore, finding a molecular marker suitable for species delimitation and for phylogenetic implications within the genus of *Stipa* is pending.

In previous studies on the tribe of Stipeae, involving representatives of *Stipa s.l*. a few nuclear markers and several plastid markers were applied. Among nuclear loci usefulness of internal transcribed spacers (ITS) was tested both as a complete ITS^15,17,20^ and separately as ITS1 and ITS2^19,21^. Moreover, external transcribed spacer (ETS) was applied in one study^19^. Recently Krawczyk *et al*.^22^ showed that the nuclear rRNA intergenic spacer region (IGS), and especially its part adjacent to 26S nrDNA, is a molecular marker giving a real chance for a phylogeny reconstruction of *Stipa*. Due to extremely high rate of evolution within the part comprising inter-repeats, the IGS region is useful for phylogenetic analyses of *Stipa* at genus level or in shallower taxonomic scale. The region seems to be the most phylogenetically informative for *Stipa* from all the chloroplast and nuclear markers tested so far^22^.

Within cpDNA utility of both, coding *(matK, ndhF, rpl16, rps3, rpoA, trnK*) and non-coding regions (*trnH-psbA, trnK-matK, trnL-trnF, trnT-trnL, rpl32-trnL, rps16-trnK* and *rps16* intron) was studied to date^15–21^. However, none of these molecular markers tested individually or as a set of loci revealed sufficient level of variability to illustrate genetic diversity within the genus of *Stipa*. Therefore, a question arises whether variability of *Stipa* representatives within plastid genome is very low, or is it concentrated in regions that had not been tested for feather grasses so far. It is also possible that the only method for species delimitation or phylogenetic inferences in *Stipa* is the use of the whole-plastid genome sequence supported by next-generation sequencing. This method, recently proposed by researches and called “super-barcoding” gives new prospects in molecular plant identification, especially in cases, where single- or multi-locus barcoding method is insufficient in discriminating closely related species^12,23–26^.

Through our research we wanted to answer the question, which or if any of plastome loci is a hotspot region for mutations and hence is useful for molecular species identification in feather grasses. For this purpose, we performed a comparative analysis of complete cpDNA sequences from 19 taxa of feather grasses to find within them the most variable DNA regions. Our analysis was conducted in two ways. In the first method, each of coding and non-coding regions was individually tested. In the second approach, we evaluated the variability of the sequence without considering its division into functional regions. We used for that a sliding window 600 bp in length moved by a 100 bp step. The length of analyzed fragment was chosen considering its potential use in DNA barcoding. In other words, we have chosen the length of a sequence that can be easily sequenced with Sanger method with a use of one pair of primers.

## Results

### Characteristics of the *Stipa* plastid genome

**Figure 1:**
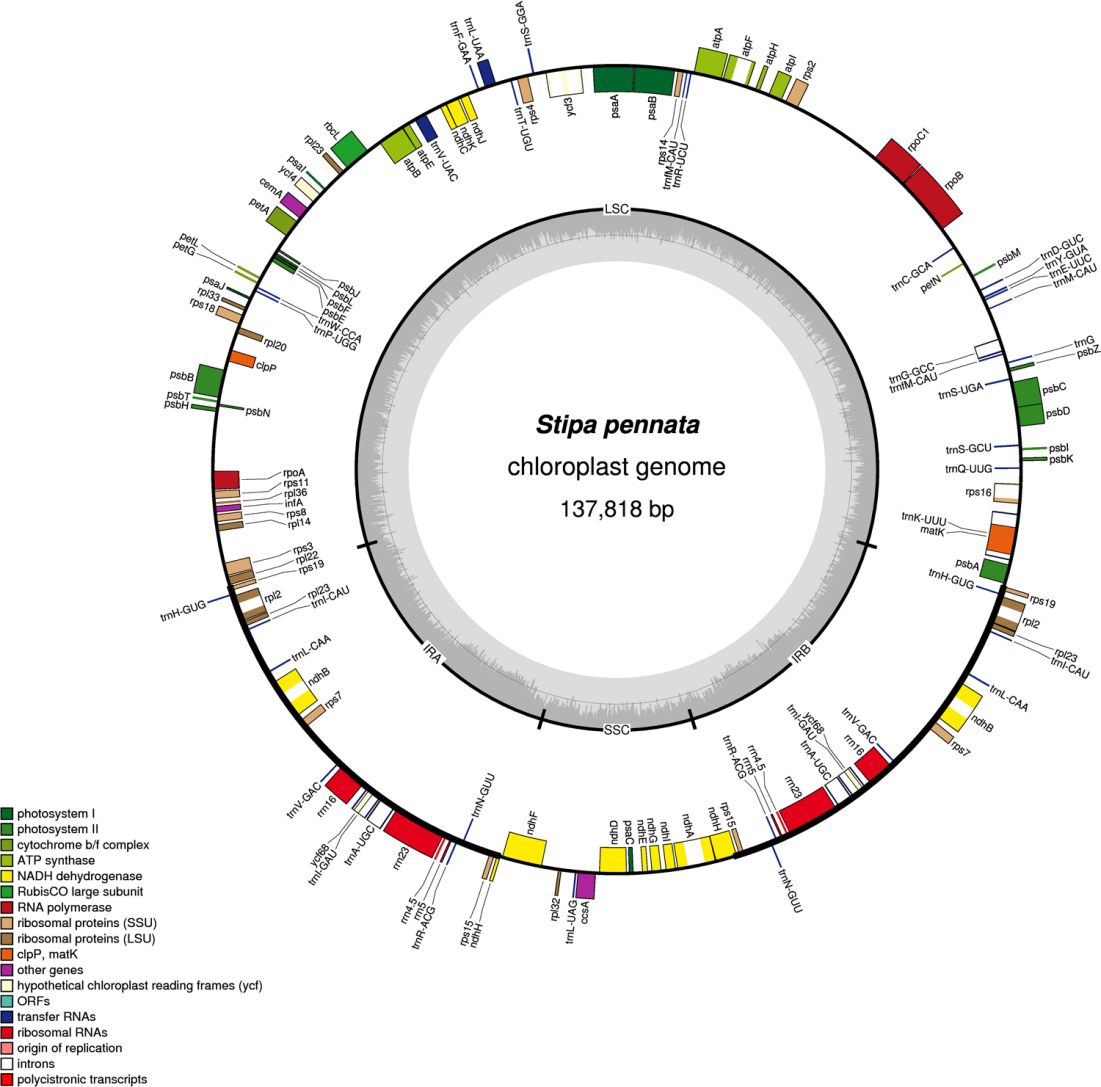
Gene map of the *Stipa pennata* subsp. *pennata* chloroplast genome. Dashed area in the inner circle indicates the GC content.

The plastome of *Stipa* was a circular molecule, comprising a large single copy (LSC) region ranging from 81,533 to 81,806 bp and a small single copy (SSC) region ranging from 12,836 to 12,837 bp, separated by two inverted repeat regions (IRs) of 21,616 bp (Fig. 1). It contained 127 genes, including 81 protein-coding genes, 8 ribosomal RNA genes and 38 tRNA genes. The genome contained 20 genes duplicated in the IRs (Fig. 1). Nine genes (*atpF*, *ndhA*, *ndhB*, *rpl2*, *rps16, trnA-UGC*, *trnG-GCC*, trnI-GAU, *trnK-UUU*) contained a single intron, while *ycf3* harbored two introns. The base composition of the genome was the following: A (30.7%), C (19.3%), G (19.5%) and T (30.5%) with an overall GC content of 38.8% and the corresponding values of the LSC, SSC and IR regions reaching 36.9, 33.0 and 44.1%, respectively.

### Sequence variation

**Figure 2:**
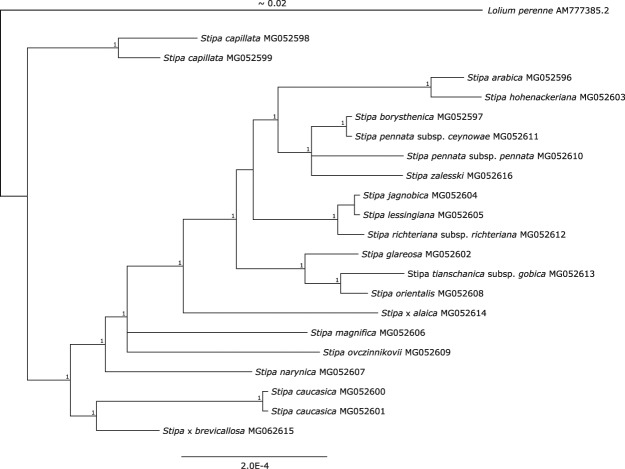
Plastome-based phylogram. The 70% majority-rule consensus phylogram derived from a Bayesian analysis of complete plastomes (excluding one Inverted Repeat region). Credibility values above 0.95 are given in the top line. For tree legibility the length of the branch of the outgroup has been shortened.

21 newly sequenced complete plastid genomes representing 19 taxa from the genus of *Stipa* were analyzed (Supplementary Table S1). Their length ranged from 137 602 bp in *S. glareosa* to 137 874 bp in *S. ovczinnikovii*. The alignment comprising the set of 21 plastomes was 138 077 bp in length and was characterized by 38.8% GC content and 99.9% pairwise identity. Sequence variability was due solely to the presence of single nucleotide polymorphism (SNP) and indels. No gene rearrangements of genome or differences in gene content were observed. The number of bases different between two compared sequences reached the highest value in the case of *S. glareosa* and *S. ovczinnikovii* and amounted to 402. At the same time, the analysis of differences between sequence pairs revealed plastomes of *S. pennata* subsp*. ceynowae* and *S. borysthenica* were identical. On a phylogram these taxa are grouped into one clade sister to *S. pennata* subsp. *pennata* and *S. zalesskii* (Fig. 2). Very few nucleotide differences were found between *S. jagnobica* and *S. richteriana* (11) as well as between *S. arabica* and *S. hohenackeriana* (17). Plastomes of two specimens representing *S. caucasica* differed in only two single indel mutations. One of them was localized in *psbH-rpoA* spacer and the second one in the *ycf3* intron. Between cpDNA sequences obtained for two representatives of *S. capillata*, 28 base differences were found. 15 of them was caused by substitutions and 13 by indel mutations. Indels were in most cases single, only in two cases comprised two adjacent nucleotides. 20 of 28 mutations were localized within intergenic spacers and three within introns. Five mutations were found within coding regions (*ndhC, rpl23, rps11, psbC* and *matK*), however only one SNP within the *psbC* gene was nonsynonymous and resulted in the replacement of Leu with Phe. In spite of differences between the specimens of *S. capillata*, they form a 100% credible clade, significantly distinct from the other representatives of the genus tested in the study (Fig. 2).

### Species delimitation

Simple heuristic search performed within The Poisson Tree Processes (PTP) method grouped the analyzed individuals with the highest support into 20 species. The result of the analysis exceeded the number of species used in the study by one, because the two representatives of *S. caucasica* were separated into two cryptic species. However, the posterior delimitation probability for both of them amounted only to 0.194 The support exceeded 0.8 only in three cases: *S. arabica* (1.0)*, S. hohenackeriana* (1.0) and *S*. × *alaica* (0.88) confirming separateness of these species with great certainty (Supplementary Table S2). Probability of delimitation for other taxa ranged from 0.247 (*S. borysthenica* and *S. pennata* subsp*. ceynowae*) to 0.795 (*S. magnifica*).

### Hotspots of variation within protein coding regions

**Figure 3:**
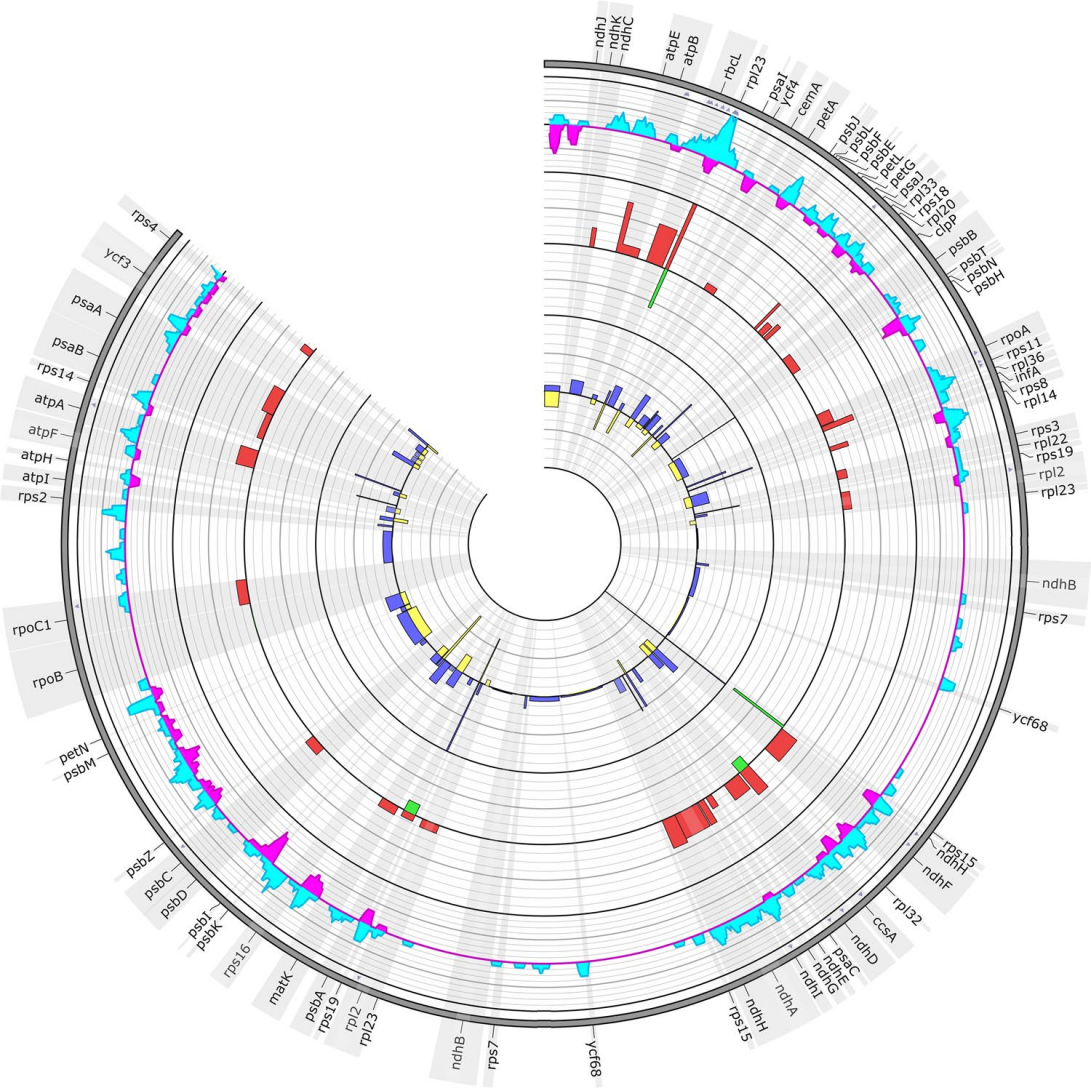
SNP and indel variation among plastomes of *Stipa*. Track A shows nonsynonymous SNP occurrence within genes. Track B and C represent identified SNPs (cyan histogram) and indels (magenta histogram) per 600 bp window size with 100 bp shift, respectively. Track D represents percent of SNPs per CDS length while track E represents percent of indels per CDS length. Track F represents percent of SNPs per noncoding region length while track G represents percent of indels per noncoding region length.

Analysis of the distribution of genetic variability within the plastome of *Stipa* revealed that the most variable protein-coding region characterized with 3.16 percent of nucleotide variation (P_V_) was one of three copies of *rpl23* gene (Fig. 3, Table 1). The copy is located near *rbcL* gene in the Large Single Copy Unit (Fig. 1) is 285 bp long (286 bp in *S. tianschanica* subsp*. gobica*) and contains eight SNP sites and one indel. In the case of *S. tianschanica* subsp*. gobica* the indel resulted in a shift of a reading frame, appearance of six internal stop codons and a pseudogenization of the gene. In the other sequences, mutations were mostly nonsynonymous and caused replacement of amino acid only once in *S. orientalis* (Pro into Gln) and twice in *S*. × *alaica* (Tyr into His). Comparative analysis of the *rpl23* gene allowed for identification of five out of 19 analyzed taxa of *Stipa*. The other two copies of the *rpl23* gene were 282 bp long and did not contain any SNPs or indels.

**Table 1:** Hotspots of variation. The most polymorphic coding and non-coding cpDNA regions over 100 bp in length and containing at least three PICs.

Region		Length [bp]	SNP	Indel	% of variation (P_V_)	π	Number of identified taxa
coding	*rpl23*	285	8	1	3.16	0.003286	5 (26.32%)
	*atpE*	414	3	0	0.72	0.001104	2 (10.53%)
	*rbcL*	1434	8	0	0.56	0.001999	3 (15.79%)
	*ndhH*	1182	5	0	0.52	0.000471	6 (31.58%)
	*ccsA*	975	4	1	0.51	0.000916	3 (15.79%)
	*ndhH*	1182	5	0	0.42	0.548000	4 (21.05%)
	*ndhA*	1089	4	0	0.37	0.000498	2 (10.53%)
	*ndhD*	1503	4	0	0.27	0.000425	2 (10.53%)
	*ndhF*	2220	6	0	0.27	0.000335	3 (15.79%)
	*atpA*	1524	4	0	0.26	0.000625	3 (15.79%)
non-coding	*rps19-psbA*	160	4	2	3.75	0.016378	2 (10.53%)
	*psbK*-*psbI*	408	1	6	1.72	0.000241	6 (31.58%)
	*ndhG-ndhI*	250	3	1	1.60	0.001157	3 (15.79%)
	*rpl33-rps18*	264	1	2	1.14	0.000364	1 (5.26%)
	*rbcL-rpl23*	274	1	2	1.09	0.003866	2 (10.53%)
	*ndhF-rpl32*	916	7	3	1.09	0.000735	7 (36.84%)
	*petA-psbJ*	822	6	2	0.97	0.002869	5 (26.32%)
	*matK-rps16*	1289	6	6	0.93	0.000580	3 (15.79%)
	*ycf4-cemA*	455	1	3	0.88	0.000244	1 (5.26%)
	*psA-ycf3*	631	4	1	0.79	0.000741	4 (21.05%)
	*rpl32-ccsA*	895	4	3	0.78	0.000523	7 (36.84%)

Among coding regions higher resolving power than *rpl23* had only the *ndhH* gene (P_V_ = 0.52%; π = 0.000471), distinguishing six (31.58%) analyzed species. However, it should be noted that the *ndhH* gene is more than four times longer than *rpl23* reaching 1182 bp in length and contains only five polymorphic sites. Even longer *rbcL* gene (1434 bp) carrying eight SNPs and characterized by P_V_ = 0.56% and π = 0.001999, successfully identified only three (15.79%) from the set of analyzed taxa.

### Hotspots of variation within non-coding regions

Among the non-coding regions, the highest frequency of polymorphism was found in 95bp-long *ndhH-ndhF* spacer (Supplementary Table S1) and in the *rps19-psbA* spacer (Table 1). Within 160 bp-long *rps19-psbA* region four SNPs and two indels were identified, which represents 3.75% of sites and is the highest value among analyzed non-coding regions over 100 bp in length. The region had also the highest π value for non-coding regions equal to 0.016378. However, the *rps19-psbA* spacer allowed to identify only two (10.53%) taxa out of the analyzed set of 19. The biggest number of taxa was successfully identified analyzing variability of *ndhF-rpl32* and *rpl32-ccsA* spacers. The percent of polymorphic sites and π score within these regions amounted to: P_V_ = 1.09%; π = 0.000735 and P_V_ = 0.78%; π = 0.000523, respectively. Relatively high variability was also characteristic for *psbK-psbI* spacer (P_V_ = 1.72%; π = 0.000241) which identified six (31.58%) taxa.

### Multi-locus barcode

Six plastome loci characterized by the highest discriminative power within *Stipa*: *ndhH*, *rpl23*, *ndhF*-*rpl32*, *rpl32-ccsA*, *psbK-psbI* and *petA-psbJ* were tested as one multi-locus barcode. It was 4508 bp long and contained 46 PICs (31 SNPs and 15 indels). Frequency of polymorphism of this barcode amounted to P_V_ = 1.44%, π score was equal to 0.001131, while the success of taxon identification reached 68.4% (13 taxa).

### Hotspots of variation within 600 bp sliding-window fragments

The analysis of 600 bp nucleotide fragments of plastome, carried out apart from their biological functions, showed that although fragments determined in this way are not characterized by the highest frequency of polymorphism, they have higher resolving power within the analyzed set of species than the regions discussed above. The biggest concentration of polymorphic sites was located in a sequence fragment comprising *rpl23* gene and adjacent regions (Fig. 3). The biggest number of taxa (10) was successfully identified by the analysis of DNA fragment comprising a part of *rbcL* gene, the complete *rbcL-rpl23* spacer and a part of *rpl23* gene (Table 2). The fragment was characterized with P_V_ = 1.67% and π = 0,003489. The percent of variation within the analyzed reading frame can be increased up to 1.83% or 2.17%, by moving the frame about 100, 200, 300 or 500 bp downwards (in 3′ direction) and by this including into analysis part of *rpl23-psaI* spacer, however discriminative power of a fragment will then decrease to nine or eight taxa (Table 2). Among the remaining 600bp-long fragments, relatively high variability was observed for a part of a *ndhF-rpl32* spacer (P_V_ = 1.33%; π = 0.000646), which identified six (31,58%) taxa. The same number of taxa was identified by the analysis of the fragment comprising *psbK* gene and adjacent parts of *trnQ-psbK* and *psbK-psbI* spacers (P_V_ = 1.33%; π = 0.000324).

**Table 2:** The most polymorphic 600 bp long regions. The number of nucleotides covered by the reading frame is given in brackets. The regions covered completely by a reading frame are given in bold.

**Comprised regions**	**Range**	**SNP**	**Indel**	**% of variation (P**_**V**_ **)**	**π**	**Number of identified taxa**
*rbcL* (16), ***rbcL-rpl23*** **(274)**, ***rpl23*** **(286)**, *rpl23-psaI* (24)	10200–10800	10	3	2.17	0.004107	9 (47.37%)
*rbcL-rpl23* (190), ***rpl23*** **(286)**, *rpl23-psaI* (124)	10300–10900	10	3	2.17	0.003489	8 (42.10%)
*rbcL* (116), ***rbcL-rpl23*** **(274)**, *rpl23* (210)	10100–10700	8	3	1.83	0.003794	9 (47.37%)
***rpl23*** **(276)**, *rpl23-psaI* (324)	10500–11100	10	1	1.83	0.001879	8 (42.10%)
*rbcL* (216), ***rbcL-rpl23*** **(274)**, *rpl23* (110)	10000–10600	7	3	1.67	0.003489	10 (52.63%)
*rbcL-rpl23* (90), ***rpl23*** **(286)**, *rpl23-psaI* (224)	10400–11000	9	1	1.67	0.001720	5 (26.32%)
*petA-psbJ* (600)	15300–15900	6	2	1.33	0.003934	5 (26.32%)
*petA-psbJ* (596), *psbJ* (4)	15400–16000	6	2	1.33	0.003934	5 (26.32%)
*rpl23* (176), *rpl23-psaI* (424)	10600–11200	8	0	1.33	0.001556	3 (15.79%)
*trnK* (41), ***trnK-rps16*** **(551)**, *rps16* (8)	95200–95800	5	3	1.33	0.001087	2 (10.53%)
*trnK-rps16* (492), *rps16* (108)	95300–95900	5	3	1.33	0.001087	2 (10.53%)
*trnK* (341), *trnK-rps16* (259)	94900–95500	4	4	1.33	0.000932	5 (26.32%)
*trnK* (241), *trnK-rps16* (359)	95000–95600	4	4	1.33	0.000932	5 (26.32%)
*ndhF-rpl32* (600)	59100–59700	5	3	1.33	0.000646	6 (31.58%)
*trnT-trnL* (588), *trnL* (12)	300–900	2	6	1.33	0.000327	4 (21.05%)
*trnT-trnL* (488), *trnL* (112)	400–1000	2	6	1.33	0.000327	4 (21.05%)
*trnQ-psbK* (113), ***psbK*** **(186)**, *psbK*-*psbI* (301)	98000–98600	2	6	1.33	0.000324	6 (31.58%)
*psbK-psbI* (407), ***psbI*** **(111)**, *psbI-trnS* (82)	98300–98900	1	7	1.33	0.000163	5 (26.32%)

## Discussion

Although examined here members of *Stipa* represents five the richest in species and well distinguished sections of the genus^3^, similarity of complete plastome sequences between pairs of species amounted from 99.7% to 100%, indicating the very low variability of the analyzed genomes. The high conservativity of the chloroplast genome in Poaceae at the generic level has previously been observed for *Bambusa*, where 18 analyzed plastomes representing different species shared 99.8% sequence similarity^27^. Slightly lower level of genome conservation reaching 96.9%-99.5% was revealed by genome-wide comparison of the *Lolium-Festuca* species complex^28^. Higher genetic variability was observed within the plastomes of dicotyledonous plants. For example, in the genus of *Daucus* complete cpDNA sequence similarity amounted to 97%^29^. In turn, in the genus *Ipomoea*, only the number of parsimony informative sites reached 3%^30^.

Analyzing the distribution of genetic variation within particular regions of plastome in *Stipa*, it can be seen that cpDNA loci previously considered in studies on this genus, do not belong to the most variable ones. Our research shows that the most polymorphic protein coding region, applied in research on *Stipa* so far^16,17^ is the *ndhF* gene. Although it is one of ten most variable genes in plastome of *Stipa*, it discriminates less than 16% of analyzed taxa. Another previously tested locus is the *trnT-trnL* spacer^18,19^. As a complete, separate region it was not characterized by a high level of variability, but under the use of 600 bp long reading frame and including part of sequence coding for *trnL*, it was among the 18 most variable and informative sequence fragments in *Stipa* (Table 2). However, our research clearly shows that neither the *ndhF* gene nor the *trnT-trnL* spacer were the most variable hotspots in plastome of the genus. Therefore, phylogenetic analyses based on them did not give satisfactory results and did not allowed for reliable phylogenetic implications.

Even lower level of genetic variability (P_V_ = 0.13%) was found within the *matK* gene (Supplementary Table S1), which is recommended as one of two core DNA barcodes for plants^31^. The *matK* region is widely used both in DNA barcoding and as a phylogenetic marker in studies on various groups of plants^12,32,33^ and was previously applied in research on *Stipa*^15–17^. The second one of the recommended and commonly used barcode loci, the *rbcL* gene^31,34,35^, although was one of three most variable coding regions in *Stipa*, its ability to discriminate between species at the level of less than 16% is highly insufficient.

The *trnL*, *matK* and *rbcL* genes were widely sequenced to develop a database of barcodes for xerothermic plants from central Europe^36^. However, a presence of only single representative of *Stipa* in this database does not allow to assess usefulness of these loci in barcoding of *Stipa*.

Another widely used as a single-loci or a component of two- or multi-locus plant barcode is *trnH-psbA*^11,12,37^. This non-coding intergenic chloroplast region exhibits extreme sequence divergence and has high rates of indels^35^. These attributes make this locus highly suitable for species discrimination^35,38^. The *trnH-psbA* spacer is especially effective barcode in pteridophytes^39^ but also in some genera of angiosperms such as *Hydrocotyle*^40^ and *Dendrobium*^41^, where the region discriminates almost all the species. Distinctive feature of the *trnH-psbA* spacer is its considerable length variation from less than 100 bp in bryophytes^42^ to more than 1000 bp in some conifers and monocots^33,43^. In the plastome of *Stipa* the *trnH-psbA* region ranged from 545 to 574 bp and contained the *rps19* gene. All the PICs (four SNPs and two indels) identified within *trnH-psbA* were located in the spacer between *rps19* and *psbA* and enabled identification only of two examined species which is very low score for such a usually variable region.

In recent years attention has been paid to high variability of the *ycf1* gene in seed plants^14,44^. Especially two regions within *ycf1* called *ycf1a* and *ycf1b*, present in the SSC region, have been predicted to have the highest nucleotide diversity (π) at the species level within angiosperm plastid genomes^44^. It has been showed that *ycf1b* generally performed better than any of the *matK*, *rbcL* and *trnH-psbA* applied individually and even was slightly better than the two-locus combination of *matK* and *rbcL*^14^. In the case of *Stipa* we were not able to assess the utility of this promising marker, because the *ycf1* locus was absent in the analyzed plastomes of feather grasses.

Analyzing the distribution of genetic diversity in cpDNA of *Stipa* and referring results of our research to the literature data, we noted that location of hot-spots for mutations within non-coding regions largely corresponds with the results obtained for monocots by Shaw *et al*.^45^, where the biggest share of PICs among non-coding regions was found in the *ndhF-rpl32* spacer. In the case of *Stipa* the region was characterized by relatively high diversity and was one of the two most successful non-coding regions in species discrimination. Among 13 most polymorphic spacers presented by Shaw *et al*.^45^ there was also the *trnT-trnL* region, discussed above, *petA-psbJ* and *trnK2-rps16*, which in *Stipa* were also in a group of most variable non-coding regions.

Spacers and introns are generally more variable than protein coding regions^46^. However, the high variability of the *rpl23* gene present in the LSC unit in *Stipa* ranks it in the third place among the most polymorphic loci just behind *ndhH-ndhF* and *rps19-psbA* spacers. The hypervariability of *rpl23* is a common phenomenon in grasses and is associated with pseudogenization of this locus. In most Poaceae there is a mutational hotspot in the region between *rbcL* and *psaI*^47,48^ containing a pseudogenized copy of *rpl23* that ranges from 40 to 243 bp in length^49^. It is supposed that *ψrpl23 locus* is a result of nonreciprocal translocation of this gene from one copy present in the inverted region^50,51^. Considering that in *Stipa rpl23* present in LSC differs in length and amino acid composition from its copies present in IRs, we can assume that the loci is also pseudogenized, and not only in *S. tianschanica* subsp*. gobica* where internal stop codons are present, but in all the studied representatives of the genus.

The results of our research indicate that the *rbcL-psaI* region together with a partial *rbcL* sequence is the best candidate barcode loci for *Stipa*. Unfortunately, even this most discriminative of the analyzed cpDNA fragments allows for identification barely 53% of taxa surveyed. The level of variation of this one, as well as any other candidate barcode region tested in this study, is too low to successfully discriminate species and to work as an effective phylogenetic marker in *Stipa*. The application of multi-locus barcode composed of six most discriminative loci allowed for identification of 68.4% of analyzed taxa, what taking into account the length of a sequence as well as the effort and cost needed for its sequencing, it is not a satisfactory result.

The problem with selecting a good barcode locus for *Stipa* results from the very low level of genetic diversity within its plastome. An extreme example is here the complete absence of nucleotide differences between cpDNA of *S. pennata* subsp. *ceynowae* and *S. borysthenica* as well as very small differences reaching 0.009% for *S. jagnobica* and *S. richteriana* and 0.012% between *S. arabica* and *S. hohenackeriana*, despite of the fact that all of the aforementioned species are morphologically well separated and easily identifiable^3,5,52^. The comparative analysis of complete cpDNA sequences as super-barcodes in the case of *Stipa* was much more effective than a traditional barcoding approach. The application of super-barcodes enabled discrimination 18 of 19 (94.74%) analyzed taxa. However, it should be noted that due to the high interspecies similarity and at the same time high variability between two representatives of *S. capillata*, the identification in some cases was based on several PICs. High conspecies variability, in some cases exceeding the congeneric variability is an unfavorable phenomenon for molecular species identification. Therefore, the species delimitation probability for *S. capillata* was low (0.296) and only slightly exceeded the support calculated for its two representatives (0.249). The analysis of dataset covered by our study provides no basis for a broader discussion on the phenomenon of barcoding gap. However, the example of *S. capillata* plastome and its in-depth comparison with cpDNA of other feather grasses shows that due to genetic diversity between its two analyzed representatives, a significant part of PICs is not specific for this species and is useless in species discrimination. It should therefore be expected that extending the dataset with multiple representatives of each species would greatly reduce the effectiveness of barcoding within *Stipa*.

In conclusion, none of the single chloroplast loci is polymorphic enough to play a role of a barcode or a phylogenetic marker for *Stipa*. Also, the effectiveness of multi-locus barcode composed of best-performing loci for *Stipa* (*ndhH*, *rpl23*, *ndhF*-*rpl32*, *rpl32-ccsA*, *psbK-psbI* and *petA-psbJ*) didn’t reach 70% of analyzed taxa. Complete plastome sequences, although applied as a super-barcode for *Stipa* are not effective in 100%, would be valuable for further study of these genus and also for a broader understanding of Poaceae plastome evolution. For molecular species identification and for phylogenetic implications within the *Stipa* genus it seems necessary to apply nuclear loci in the studies.

## Materials and Methods

### Plant material

Plant material of *Stipa* taxa used in the study was collected during the field studies in 2010–2016 (Supplementary Table S1). 21 individuals representing 19 taxa from the genus of *Stipa* were analyzed. Only in few cases material (dry leaves) for molecular studies were taken from older herbarium specimens. Samples of all the taxa used in the analyses are preserved at KRA (Herbarium of the Institute of Botany, Jagiellonian University).

### Plastid genome sequencing and assembly

A genomic library for MiSeq sequencer was developed with the use of the Nextera XT Kit (Illumina, San Diego, CA, USA) according to the manufacturer protocol. The libraries were quantified and normalized using Kappa Library Quantification Kit for Illumina (Kapa, Wilmington, MA, USA) and sequenced using the MiSeq. 600v2 cartridge that enable 2x300 bp pair-end reads. The details on library preparation, validation, quantification and sequencing are given in Myszczyński *et al*.^53^ and in Sawicki *et al*.^54^. The remained libraries were prepared using TruSeq Nano DNA Library Preparation Kit (Illumina) and sequenced using HiSeq. 2500 sequencer (Illumina) by Macrogen Inc. (Korea).

The obtained raw reads were cleaned by removing low quality and short (<than 50 bp) reads. The remained reads were assembled de novo using Velvet assembler as implemented in the Geneious R8 software (Biomatters, Auckland, New Zealand).

The flow chart for the sin silico reconstruction of the sequenced *Stipa* plastomes was the same as previously published^55^.

### Confirmation of plastome structure through nanopore sequencing

The junctions between single-copy and inverted repeats regions were confirmed by long-read nanopore sequencing. Genomic library was prepared using Rapid Sequencing Kit R9 Version SQK RAD001 (Oxford Nanopore Technologies, UK). The library was sequenced using SQK-MAP005 Nanopore Sequencing Kit, Spot-ON Flow Cell Mk1 and MinION Mk1B device (Oxford Nanopore Technologies, UK). The reads were assembled using Canu software^56^.

### Annotation and construction of a physical map of the plastome

The annotation of chloroplast genome was conducted using the Geneious R9 software (Biomatters, Auckland, New Zealand) by comparing with the genome of *Stipa lipskyi* (GenBank accession no. KT692644)^53^. A physical map of the plastome was generated using OGDRAW 1.2 (http://ogdraw.mpimp-golm.mpg.de)^57^.

### Variation analyses

Chloroplast genomes of 21 taxa of *Stipa* genus were aligned using the MAFFT genome aligner^58^. Afterwards based on alignment of genomes polymorphism analyses were conducted separately for each coding sequence, intron and intergenic spacer. Every variation within aforementioned regions was identified as single nucleotide polymorphism (SNP) or insertion/deletion (indel) and counted using custom Python script. Each SNP within coding sequence was tested if it affects the protein sequence and defined as synonymous or nonsynonymous SNP. Finally, variations were visualized using Circos software^59^ combined with custom Python script.

### Phylogenetic analyses

Phylogenetic analyses were performed using 21 aforementioned *Stipa* taxa and *Lolium perenne* L. as a root species. First, PartitionFinder2^60^ was used to determine the best partitioning schemes and corresponding nucleotide substitution models. The data-set blocks were predefined a priori based on protein coding genes (CDS) and intergenic spacers as well as for first, second and third position for each of CDS. The Bayesian information criterion (BIC) and the ‘greedy’ algorithm with branch lengths estimated as unlinked were used to search for the best-fit scheme. Phylogenetic analyses were conducted using BI method. Bayesian analysis (BI) was conducted using MrBayes 3.259^61^, and the MCMC algorithm was run for 20,000,000 generations (sampling every 1,000) with four incrementally heated chains (starting from random trees). The first 1000 trees were discarded as burn-in, and the remaining trees were used to develop a Bayesian consensus tree.

### Species delimitation

The Poisson Tree Processes method was applied to delimitate species boundaries^62^. The analysis was performed using a rooted tree, the MCMC algorithm was run for 500 000 generations, with 100 thinning and 0.2 burn-in.

## Electronic supplementary material

Supplementary Table S1 and Supplementary Table S2
