# Supplementary material for: Plastid super-barcodes as a tool for species discrimination in feather grasses (Poaceae: Stipa)
Source: Sci Rep. 2018 Jan 31;8:1924. doi: 10.1038/s41598-018-20399-w (PMC5792575; doi:10.1038/s41598-018-20399-w)
Supplement: Supplementary file 1 — Supplementary Table S1 and Supplementary Table S2 [file 41598_2018_20399_MOESM1_ESM.pdf]

Title: **Plastid super-barcodes as a tool for species discrimination in feather grasses (Poaceae: *Stipa*)**

Authors: Katarzyna Krawczyk, Marcin Nobis, Kamil Myszczyński, Ewelina Klichowska, Jakub Sawicki

Supplementary Information

Supplementary Table S1. Specimens used in the study, sequence length and GenBank accession numbers.

Taxon	Section	Country	Voucher	Sequence length [bp]	GB accession number
Stipa × *alaica* Pazij	*Smirnovia*	Kyrgyzstan	40 km W of Sary-Tash, 2942 m, 3 Jul 2015, *M. Nobis, A. Nowak* (KRA); S/7/10	137 850	MG052614
S. arabica Trin. & Rupr.	*Barbatae*	Kyrgyzstan	Toktogul, 5 July 2013, *M. Nobis & A. Nowak* (KRA); S/61/4	137 757	MG052596
S. borysthenica Klok.	*Stipa*	Poland	Gryfino, Dolna Odra, *A. Popiela* (SZUB); S/74/3	137 825	MG052597
S. × *brevicallosa* M. Nobis	*Smirnovia*	Tajikistan	Zeravshan Mts, Mogien settl., 25 May 2015, *M. Nobis, A. Nowak* (KRA); S/15/4	137 850	MG052615
S. capillata L.	*Leiostipa*	Russia	Tyva Resp., 9 Jul 2007, *P. Shavrova* (KRA); S/4/2	137 830	MG052598
S. capillata L.	*Leiostipa*	Georgia	10 km N of Akhalkalaki (on road to Bakyriani), 27 Jul 1982, *Rusanovich* (KRA); S/4/3	137 835	MG052599
S. caucasica Schmalh.	*Smirnovia*	Kazakhstan	150 km W of Almaty, 18 May 2014, *M. Nobis, P. Gudkova* (KRA); S/53/1	137 798	MG052600
S. caucasica Schmalh.	*Smirnovia*	Kyrgyzstan	Dzhel-Aryk, 16 Jun 2013, *M. Nobis, A.*	137 800	MG052601

			Nowak (KRA); S/53/4		
S. glareosa P.A. Smirn.	*Smirnovia*	Russia	Baical, 21 Jul 2013, *A.L. Ebel* (KRA); S/49/9	137 602	MG052602
S. hohenackeriana Trin. & Rupr.	*Barbatae*	Tajikistan	Zeravshan Mts, Mogien, 27 May 2015, *M. Nobis, A. Nowak* (KRA); S/13/2	137 753	MG052603
S. jagnobica Ovcz. & Czuk.	*Leiostipa*	Tajikistan	0.5 km E of Serimadarun Lake, 2320 m, 30 May 2015, *M. Nobis*; (KRA); S/9/4	137 827	MG052604
S. lessingiana Trin. & Rupr.	*Subbarbatae*	Kazakhstan	40 km NW of Almaty, 690 m a.s.l., 22 May 2014, *M. Nobis, P. Gudkova* (KRA); 460167	137 829	MG052605
S. magnifica A. Junge	*Smirnovia*	Kyrgyzstan	Tian-Shan Mts, Tashkumyr, 650 m, 11 May 2011, *M. Nobis, A. Nowak* (KRA); S/21/1	137 848	MG052606
S. narynica M. Nobis	*Smirnovia*	Kyrgyzstan	E of Tashkumyr, 11 May 2011, *M. Nobis, A. Nowak* (KRA); S/74/1	137 854	MG052607
S. orientalis Trin.	*Barbatae*	Tajikistan	Zeravshan Mts, near Iskanderkul Lake, 2300 m, 15 June 2011, *M. Nobis* (KRA); S/24/2	137 823	MG052608
S. ovczinnikovii Roshev.	*Smirnovia*	Tajikistan	Zeravshan Mts, Vashan River valley, 1 Jun 2015, *M. Nobis* (KRA); S/17/7	137 874	MG052609
S. pennata subsp. *ceynowae* Klichowska & M. Nobis	*Stipa*	Poland	Folusz settl., May 2015, *E. Klichowska* (KRA); S/72/1	137 825	MG052611
S. pennata L. subsp. *pennata*	*Stipa*	Poland	Skarpy Ślesińskie near Slesin, May 2015, *E. Klichowska* (KRA); S/42/16	137 818	MG052610

S. richteriana Kar. & Kir. subsp. *richteriana*	*Leiostipa*	Kazakhstan	Balkhash, 22 May 2014, *M. Nobis* (KRA); S/70/1	137 831	MG052612
S. tianschanica Roshev. subsp. *gobica* (Roshev.) D.F. Cui	*Smirnovia*	Mongolia	Iche Bogdo, 27 Jul 1974, *A. Pacyna* (KRA); S/78/2	137 847	MG052613
S. zalesskii Wlenskii	*Stipa*	Kyrgyzstan	12 km NW of Sassumyr, 7 Jul 2015, *M. Nobis, A. Nowak* (KRA); S/83/1	137 836	MG052616

Supplementary Table S2. Species delimited from set of analyzed individuals by PtP simple heuristic search.

Species delimited by PtP analysis	Posterior delimitation probability
Stipa × *alaica*	0.880
S. arabica	0.998
S. borysthenica	0.247
S. × *brevicallosa*	0.375
S. capillata	0.296
S. caucasica	0.194
S. caucasica (Kazakhstan)	0.194
S. caucasica (Kyrgyzstan)	0.194
S. glareosa	0.640
S. hohenackeriana	0.996
S. jagnobica	0.336
S. lessingiana	0.336
S. magnifica	0.795
S. narynica	0.629
S. orientalis	0.329
S. ovczinnikovii	0.711
S. pennata subsp. *ceynowae*	0.247

S. pennata subsp. *pennata*	0.730
S. richteriana subsp. *richteriana*	0.656
S. tianschanica subsp. *gobica*	0.329
S. zalesskii	0.483
